# Seafarers’ Occupational Noise Exposure and Cardiovascular Risk. Comments to Bolm-Audorff, U.; et al. Occupational Noise and Hypertension Risk: A Systematic Review and Meta-Analysis. *Int. J. Environ. Res. Public Health* 2020, *17*, 6281

**DOI:** 10.3390/ijerph18031149

**Published:** 2021-01-28

**Authors:** Lucas David, Loddé Brice, Pougnet Richard, Dewitte Jean Dominique, Jégaden Dominique

**Affiliations:** 1 ORPHY Laboratory, University Brest, F-29200 Brest, France; brice.lodde@chu-brest.fr; 2 Occupational and Environmental Diseases Center, Teaching Hospital, F-29200 Brest, France; richard.pougnet@chu-brest.fr (P.R.); jean-dominique.dewitte@chu-brest.fr (D.J.D.); 3 French Society of Maritime Medicine Brest, F-29200 Brest, France; dominique.jegaden@wanadoo.fr; 4 Laboratoire d’Etude et de Recherche en Sociologie (EA 3149), Université de Brest—Bretagne Occidentale, F-29200 Brest, France

A German team published a new meta-analysis on the topic, “Occupational noise and hypertension risk: A systematic review and meta-analysis” in the International Journal of Environmental Research and Public Health [1]. They conclude to the relationship between a high level of blood pressure and noise exposure. One of the 23 selected articles was published in French in 1986 by Jégaden et al. It involved 455 seafarers aged from 40 to 55—164 mechanics and 291 deck crew members [2]. There is a relation between existence of lower auditiv capacity for the 4000 Hz frequency and exposure upper than 85 dB(A) [3]. The various impact of noise exposure level between these two categories of seafarers was only noticed among the mechanics’ strength. The prevalence of confirmed hypertension among mechanics was 18.90% (12.8–25%) compared to 11.68% (7.92–15.44%) in deck crew members (*p* < 0.05). The relative risk was 1.62 (1.03–2.53) and was independent to other risk factors of hypertension (obesity, alcohol consumption, hypertensive heredity). It also indicated that the occurrence of hypertension was related, on the one hand, to a high level of noise (>85 dB(A)) and, on the other hand, to a duration of exposure to noise for more than 20 years [2]. Similar to other publications, Bolm–Audorff meta-analysis provides clear evidence of the relationship between exposure to industrial noise above 85 dB(A) and the risk of high blood pressure [4,5,6,7]. This relative risk is calculated at 1.72 (1.48–2.01) [1]. Fu et al. find a relative risk of 1.62 (1.40–1.88) and Skogstad et al. 1.68 (1.10–2.57), similar to Jégaden et al. [2,4,5]. In a contradictory way, some studies continue to fail to find this relationship, including marine environment [8,9].

Physical stress such as noise, vibrations, and also psychological (stress, confinement, isolation, boredom) or, recently with the COVID-19 pandemic, infectious damage in confined environments are a natural part of a seafarer’s environment and probably have a collateral impact on their health. The inclusion in this recent meta-analysis of Jégaden’s work highlights that the marine environment could be considered as a model to study human reactions to stress.

A recent meta-analysis on the impact of noise on the cardiovascular system concluded that living or working in an environment with noise exposure is associated with an increased risk of Cardiovascular mortality (HR 1.12; CI 95% 1.02–1.24) [5]. Pathophysiological mechanisms of the long-term effects of noise on the cardiovascular system include unexpected consequences: heart rate variability, oxidative stress and vascular dysfunction due to alteration of the autonomic nervous system [10]. According to the Babish’s noise effect models, exposure to noise influences the ANS either directly or indirectly through the stress hormones [11]. Indeed, chronic stress due to a “fight-or-flight” response could generate increased blood pressure, lipids or glucose levels and the activation of blood coagulation [12,13].

Over the past 40 years, risk prevention has significantly decreased ship noise levels (improved insulation of living spaces, automation of machines, soundproof monitoring rooms, diesel–electric propulsion, etc.). Tu and Jepsen report that “Measured noise levels are highest in the engine rooms, followed by the levels on deck. This may partly explain the high prevalence of hypertension for seafarers working in these two areas.” [14]. In his recent register-based longitudinal cohort study of 85,169 Swedish seafarers, Erikson found significant increased mortality in male deck crew and engine crew. A significant difference with passenger ferries’ crew members was noticed. One hypothesis that they still discussed in a previous article is the level of noise exposure [15,16]. On the contrary, Oldenburg does not discuss noise as a cause of high blood pressure in seafarers [17]. However, we must remain vigilant among fishermen, many of whom are still exposed to a large amount of noise and remember that this occupational exposure is added to many other risks of hypertension, such as obesity, sedentary lifestyle and alcoholism. Few studies on cardiovascular risk and disease incidence have been published. The need of cohort and cross-sectional studies in maritime industry workers is urgent to better prevent cardiovascular diseases; As Pougnet et al. said, “Are we underestimating the cardiovascular risks of seafarers?” [18].
